# Detection of Circulating Cancer‐Associated Fibroblasts in Head and Neck Squamous Cell Carcinoma and Their Impact on Circulating Tumor Cells

**DOI:** 10.1002/hed.28239

**Published:** 2025-07-05

**Authors:** Kazuaki Chikamatsu, Hideyuki Takahashi, Shota Ida, Hiroe Tada, Miho Uchida, Masaomi Motegi, Yuichi Tomidokoro

**Affiliations:** ^1^ Department of Otolaryngology‐Head and Neck Surgery Gunma University Graduate School of Medicine Maebashi Gunma Japan

**Keywords:** circulating cancer‐associated fibroblast, circulating tumor cell, EpCAM, head and neck squamous cell carcinoma, liquid biopsy

## Abstract

**Background:**

Like tumor cells, cancer‐associated fibroblasts (CAFs) can enter the bloodstream and may function in systemic circulation.

**Methods:**

Circulating CAFs (cCAFs) were isolated from the peripheral blood of head and neck squamous cell carcinoma (HNSCC) patients using CD45 depletion and detected by *FAP* expression using RT‐qPCR. Circulating tumor cells (CTCs) were detected through the expression of epithelial markers (*EPCAM*, *EGFR*, and *MET*). We analyzed the association between cCAF positivity and clinical factors, CTC characteristics, and verified tumor tissue relationships using public datasets.

**Results:**

Of the 97 patients, 19.6% were positive for cCAFs and 61.9% for CTCs. cCAF positivity was significantly associated with lymph node metastasis, CTC positivity, and lower *EPCAM* levels on CTCs. In tumor tissues, *FAP* expression inversely correlated with *EPCAM* expression in tumor cells.

**Conclusion:**

cCAFs may contribute to the survival of CTCs and tumor progression through changes in the characteristics of CTCs.

## 1 Introduction

Accumulating evidence has indicated that circulating tumor cells (CTCs) shedding from the primary tumors into the bloodstream play an important role in the development of distant metastasis as well as locoregional recurrence [1, 2, 3]. Thus, CTCs serve as one of the potential biomarkers in liquid biopsy, a tool for diagnosing and monitoring cancer. In head and neck squamous cell carcinoma (HNSCC), the detection and clinical significance of CTCs have been extensively investigated [4, 5]. Our previous report has revealed that CTC positivity was significantly correlated with treatment response, recurrence, and prognosis [6]. Similar findings have been already validated by various studies [7]. Furthermore, the analysis of the genomic, molecular, and biological characteristics of CTCs has provided new insights into their clinical significance and has also contributed to elucidating mechanisms involved in recurrence, metastases, and immunosuppression.

On the other hand, CTCs are exposed to harsh environments including hemodynamic shear forces, collision with leukocytes, induction of anoikis by detachment from the extracellular matrix, and immune surveillance, and therefore, CTCs are known to have a short half‐life in systemic circulation [8]. Besides forming clusters for survival advantages, CTCs seek to survive through interaction with surrounding cells even in the bloodstream. On the contrary, CTCs can acquire further malignant traits such as epithelial mesenchymal transition (EMT), stemness, immune suppression, and treatment resistance. For instance, platelets guard tumor cells by forming heteroaggregates from immune elimination and support attachment to the endothelium within the circulatory system, promoting the establishment of hematogenous metastasis [9]. Moreover, CTCs are driven in cell cycle progression through the interaction with neutrophils, thereby accelerating metastatic spread [10]. In addition to the earlier, the survival strategy of CTCs in the blood microenvironment is a complex process mediated by various factors [11]; however, their mechanisms have yet to be fully elucidated.

Cancer‐associated fibroblasts (CAFs), which are one of the major components in the tumor microenvironment (TME), are well‐known to contribute to tumor growth, invasion, metastasis, and immune evasion through dynamic crosstalk with tumor cells [12]. Since CAFs display high motility and migration ability compared to normal fibroblasts [13, 14]. Like tumor cells, CAFs also have the potential to shed into the bloodstream and may function in systemic circulation. CAF progenitor cells, mesenchymal stem cells, are recruited from bone marrow to the stroma of tumor tissues [15, 16]. Recent findings have revealed that CAFs exist in the blood circulation in cancer patients and that their presence is involved in the characteristics of CTCs by forming aggregate clusters and has clinical implications [17, 18, 19].

Herein, we first investigated the presence of circulating CAFs (cCAFs) in peripheral blood obtained from patients with head and neck squamous cell carcinoma (HNSCC), and then further analyzed the clinical significance of cCAFs and their impact on CTCs. Our findings provide a new insight into understanding the interplay between cCAFs and CTCs as well as the potential clinical application as a tool for liquid biopsy.

## 2 Materials and Methods

### 2.1 Patient and Blood Collection

Peripheral blood samples were collected from 20 healthy donors and 23 patients with HNSCC in addition to the 74 previously analyzed patients [20]. Blood samples were collected from the middle of the vein in order to avoid contamination with epithelial cells and stromal cells from the skin, as described previously [6]. Patients did not receive any anticancer drugs, radiotherapy, immunotherapy, or surgery before blood collection. Clinical information, including age, sex, primary site, T classification, N classification, M classification, stage, and relapse, was collected from medical records. This study was approved by the Institutional Review Board of Gunma University (HS2017‐152). Written informed consent was obtained from each patient.

### 2.2 Isolation of CAFs From Tumor Tissues

CAFs were isolated from surgical specimen of a patient with HNSCC according to a previously described protocol [21]. Briefly, tumor tissues sliced into 1–2 mm pieces were plated in six‐well tissue culture plates with DMEM supplemented with 10% fetal calf serum, 100 units/mL penicillin, and 100 μg/mL streptomycin (all reagents from Gibco, Grand Island, NY) and cultured until the sufficient outgrowth of cells. After culturing cells through several passages, fibroblasts were confirmed by the detection of fibroblast activation protein (FAP), CD90, and α‐smooth muscle actin (α‐SMA) using flow cytometry as previously described and were used for preliminary experiments.

### 2.3 Isolation of cCAFs and CTCs

cCAFs and CTCs were isolated from peripheral blood samples using the CD45 depletion method, as described previously [20]. Briefly, peripheral blood mononuclear cells (PBMCs) isolated by density gradient technique were depleted of contaminating red blood cells using red blood cell lysis buffer (Roche Diagnostic GmbH, Mannheim, Germany), and cCAFs and CTCs were then purified using the EasySep Human CD45 Depletion Kit II (STEMCELL Technologies, Vancouver, Canada) according to the manufacturer's protocols. The concentrated cCAFs and CTCs were used for further experiments.

### 2.4 Gene Expression Analysis

Gene expression analysis was performed as previously described [20]. Total RNA was extracted from samples containing cCAFs and CTCs using the RNeasy micro kit (Qiagen, Hilden, Germany) according to the manufacturer's protocols. cDNA was synthesized using the QuantiTect Reverse Transcription kit (QIAGEN), and preamplification was performed using the TaqMan PreAmp Master Mix kit (Thermo Fisher Scientific, Waltham, MA, USA). The preamplified cDNA samples were analyzed using real‐time quantitative polymerase chain reaction (PCR) (Thermo Fisher Scientific) for gene expression. Expression levels of three epithelial‐related genes (*EPCAM*, *EGFR*, and *MET*) and three CAF‐related genes (*FAP*, *ACTA2*, and *PDGFRA*) were determined. On detecting at least one of the three epithelial‐related genes, the sample was defined as CTC positive. *ACTB* was used as a control. All primers were obtained from Thermo Fisher Scientific (TaqMan Gene Expression Assays), and data regarding the seven PCR primers used are listed in Table S1. Baseline expression of *ACTA2* and *PDGFRA* genes was determined using the average Ct value of 20 healthy donor samples, and expression levels of the target genes in cCAFs were assessed as the fold change compared with that in healthy donor samples using the relative quantification 2^–∆∆Ct^ method.

### 2.5 Acquisition of the GSE164690 Dataset From a Publicly Available Database

The GSE164690 dataset, which includes single‐cell RNA sequencing (scRNA‐seq) data, was downloaded from the Gene Expression Omnibus (GEO) database (http://www.ncbi.nlm.nih.gov/geo/). CD45‐negative cells isolated from 15 freshly resected HNSCC tumors were analyzed using the Seurat version 4 R package. Cells that have below 100 expressed genes were removed. After a global‐scaling normalization, 2000 features that exhibit high cell‐to‐cell variation were calculated for downstream analyses. Nonlinear dimensional reduction was performed using the Uniform Manifold Approximation and Projection (UMAP). Cells were clustered using the FindCluster function, and the cell type of each subcluster was defined by differentially expressed genes.

### 2.6 Statistics Analysis

The data were analyzed with GraphPad Prism 8.0 (GraphPad Software, San Diego, CA, USA). The unpaired *t* test was used for continuous variables, whereas the chi‐squared test and Fisher's exact test were employed for categorical variables. Survival curves were plotted using the Kaplan–Meier method, and the log‐rank test was used to compare survival curves between groups. The correlations of two continuous variables, gene expression levels, were determined by the Pearson's correlation coefficient. Two‐sided *p*s < 0.05 were considered statistically significant.

## 3 Results

### 3.1 Preliminary Experiments for Detection of cCAFs

**FIGURE 1:**
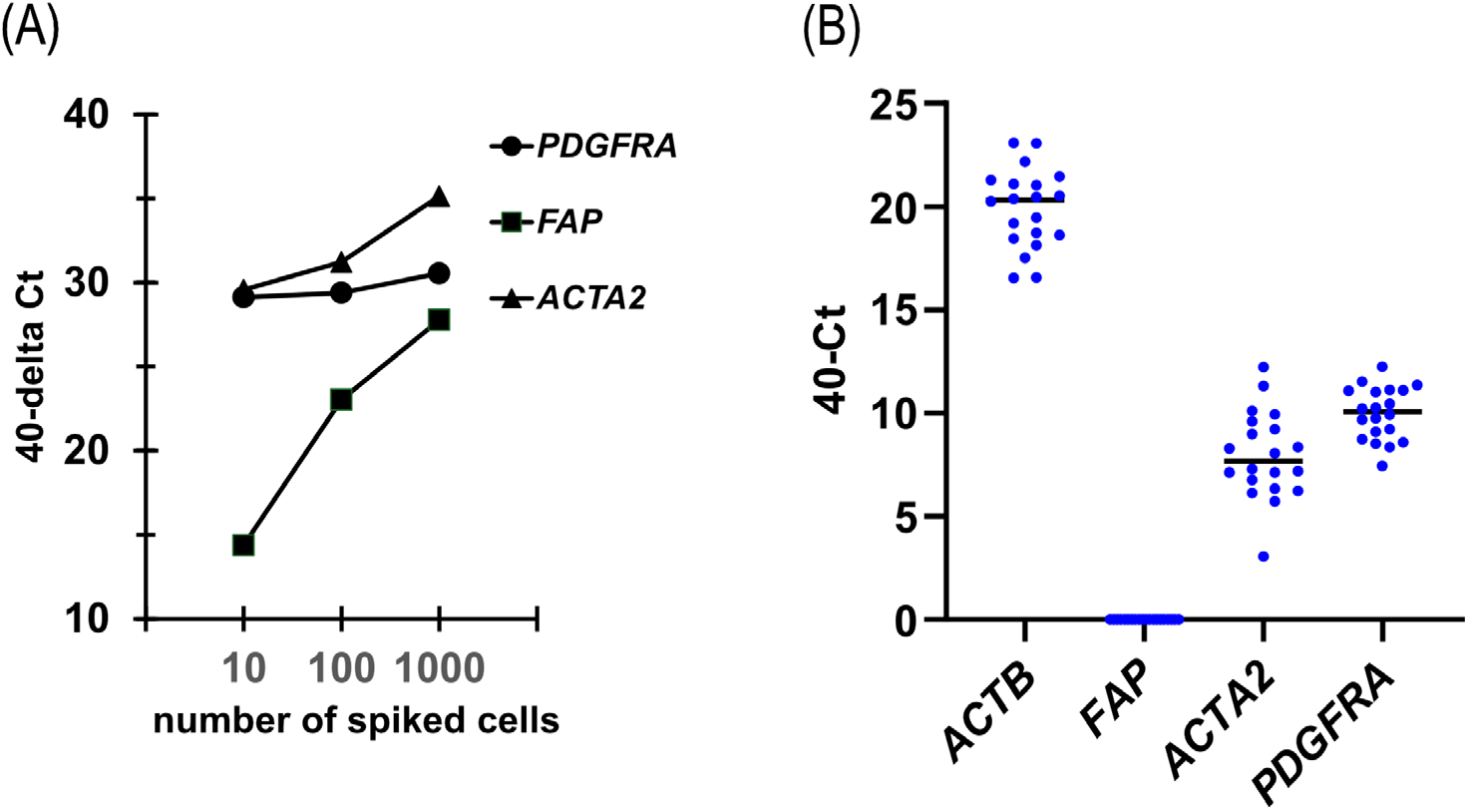
Detection of cancer‐associated fibroblasts (CAFs) in preliminary experiments. (A) Standard curve of gene expression calculated from serial dilutions of CAFs using real time RT‐qPCR. (B) Expression of four genes, *ACTB*, *FAP*, *ACTA2*, and *PDGFRA* in samples obtained from 20 healthy donors. When the cycle threshold (Ct) was not determined, Ct value is calculated as 40. [Color figure can be viewed at wileyonlinelibrary.com]

A preliminary experiment was conducted to confirm whether cCAFs in the peripheral blood of patients with HNSCC were identified by molecular detection techniques and which markers would be suitable for cCAF detection. We spiked a low number of CAFs (10, 100, and 1000 cells) into 7.5 mL of whole blood from a healthy donor. The spiked blood sample was processed in the same way as the patient samples, and mRNA extraction, cDNA synthesis, preamplification, and RT‐qPCR were performed. Among the three CAF‐related markers, though the gene expression level of *FAP* and *ACTA2* showed a dose‐dependent fashion, the expression of *PDGFRA* remained almost constant regardless of the number of spiked CAFs (Figure 1A). We next investigated the gene expression from samples obtained from 20 healthy donors. As shown in Figure 1B, the *FAP* gene was not detected in any of the 20 cases. Meanwhile, *ACTA2* and *PDGFRA*, as well as *ACTB*, were detected in all 20 cases. Based on these results, we selected *FAP* gene expression as an identification marker for cCAFs in peripheral blood.

### 3.2 Detection of cCAFs and CTC in Patients With HNSCC

**FIGURE 2:**
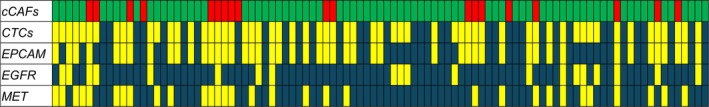
Heatmap depicting detection of circulating cancer‐associated fibroblasts and circulating tumor cells and the three epithelial‐related gene expressions in samples obtained from patients with HNSCC. The red or yellow and green or navy blue squares denote positive and negative, respectively. [Color figure can be viewed at wileyonlinelibrary.com]

**FIGURE 3:**
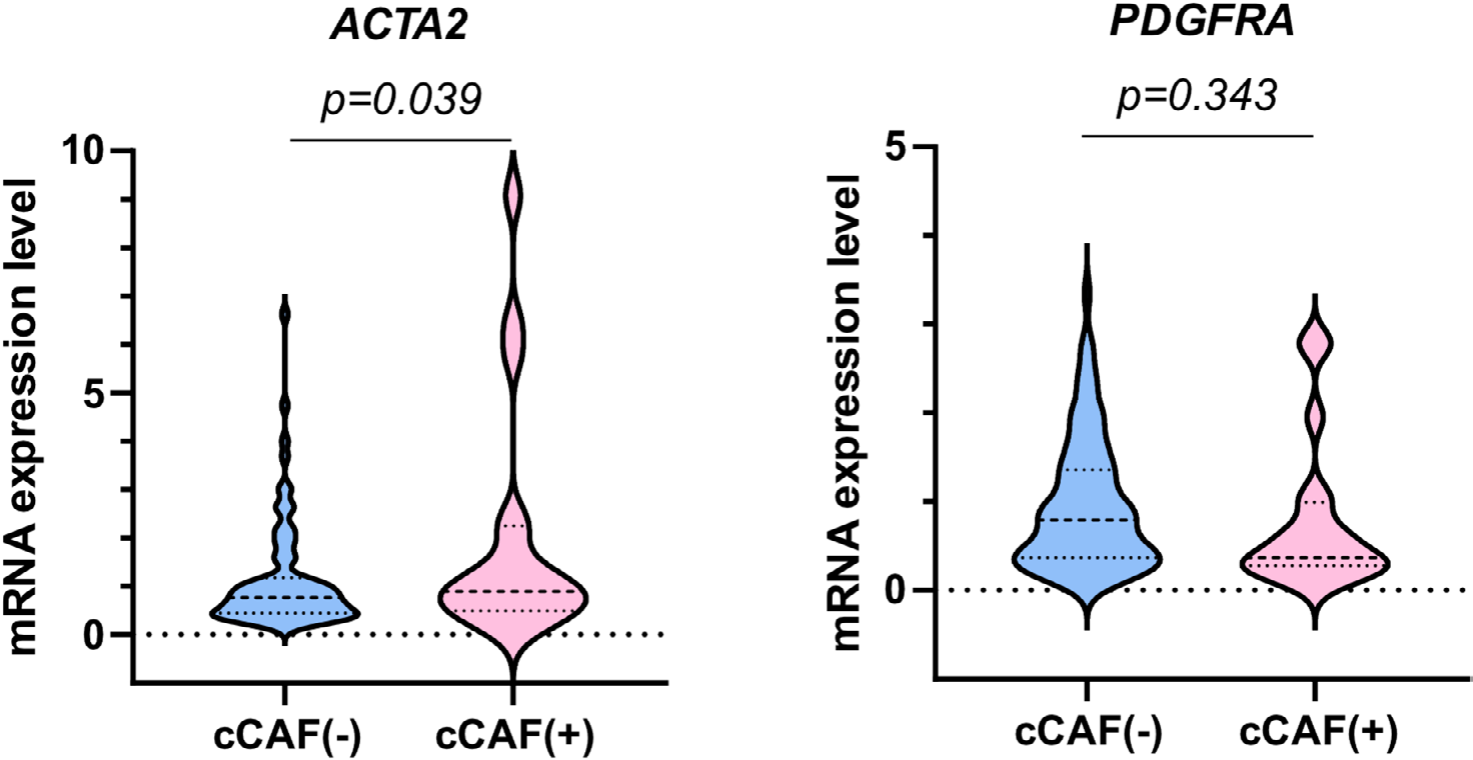
Comparison of expression levels of *ACTA2* and *PDGFRA* genes between HNSCC patients with and without cCAFs. [Color figure can be viewed at wileyonlinelibrary.com]

A total of 97 patients with HNSCC was investigated for the presence of cCAFs and CTCs in peripheral blood (Figure 2). Nineteen (19.6%) and 60 (61.9%) of the 97 patients with HNSCC were positive for cCAFs and CTCs, respectively. Of the two CAF‐related genes that were not markers for cCAFs identification, the expression level of *ACTA2* was significantly higher in patients with cCAFs (Figure 3), suggesting that *ACTA2* gene expression in samples may partly reflect that expressed in cCAFs. Among the 60 CTC‐positive patients, 48 (80.0%) were *EPCAM*‐positive, 20 (33.3%) were *EGFR*‐positive, and 31 (51.7%) were *MET*‐positive (Figure 2).

### 3.3 Relationship Between the Presence of cCAFs and Clinical Factors

The relationship between the presence of cCAFs and clinical factors is summarized in Table 1. The patients in whom cCAFs were identified showed a significantly higher rate of lymph node metastasis. Most notably, the patients in whom cCAFs were identified also had a significantly higher proportion of CTC‐positive patients. On the other hand, there was no significant correlation with clinical outcomes (Figure S1A,B). We further analyzed whether the existence of cCAFs along with CTCs in the circulation had prognostic implications; however, both CTC and cCAF existence did not significantly affect prognosis (Figure S1C,D).

**TABLE 1:** Patient demographics and clinical characteristics.

Clinical variable		cCAF (+)	cCAF (−)	*p*.
		*n* = 19	*n* = 78	
Age Median (range) 68 (39–89)	< 68	10	37	0.7995
	≥ 68	9	41	
Sex	Male	19	70	0.3488
	Female	0	8	
Primary site	Oral cavity	0	6	0.3287
	Oropharynx	11	41	
	Hypopharynx	6	15	
	Larynx	2	16	
HPV status	Positive	8	37	0.7992
	Negative	11	41	
T factor	T0‐2	6	39	0.2012
	T3‐4	13	39	
N factor	N0	1	24	0.0214
	N1‐3	18	54	
M factor	M0	18	71	> 0.999
	M1	1	7	
Stage	I–II	5	36	0.1302
	III–IV	14	42	
Relapse	(+)	9	30	0.603
	(−)	10	48	
Locoregional recurrence	(+)	4	18	> 0.999
	(−)	15	60	
Distant metastasis	(+)	5	13	0.3366
	(−)	14	65	
CTC	(+)	16	44	0.0342
	(−)	3	34	

*Note:* Bold values indicate statistically significant differences (*P* < 0.05).

Abbreviations: cCAF: circulating cancer‐associated fibroblast, HPV: human papillomavirus, CTC: circulating tumor cell.

### 3.4 Relationship Between the Presence of cCAFs and Epithelial‐Related Gene Expression in CTCs

**FIGURE 4:**
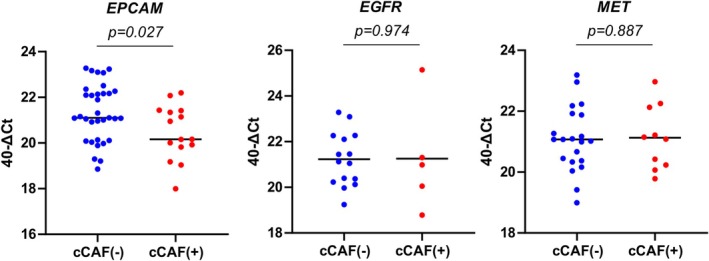
Comparison of expression levels of epithelial‐related genes, *EPCAM*, *EGFR*, and *MET*, in circulating tumor cells (CTCs) between patients with HNSCC with and without circulating cancer‐associated fibroblasts. [Color figure can be viewed at wileyonlinelibrary.com]

We next analyzed whether the presence of cCAFs is associated with molecular phenotypes of CTCs. cCAF‐positive patients had a significantly higher proportion of *EPCAM*‐positive CTCs, but not *EGFR*‐positive or *MET*‐positive CTCs, compared with cCAF‐negative patients (Table 2). Interestingly, the expression level of *EPCAM* in CTCs was, conversely, decreased in cCAF‐positive patients (Figure 4). These results suggest that while cCAFs support the survival of *EPCAM*‐positive CTCs, they may also potentially induce phenotypic changes in CTCs.

**TABLE 2:** Correlation of the presence of cCAFs and epithelial‐related marker expression on CTCs.

Epithelial‐related markers	Expression	cCAF (+)	cCAF (−)	*p*
		*n* = 19	*n* = 78	
*EPCAM*	Negative	4	45	0.0049
	Positive	15	33	
*EGFR*	Negative	14	63	0.5320
	Positive	5	15	
*MET*	Negative	9	57	0.0524
	Positive	10	21	

*Note:* Bold values indicate statistically significant differences (*P* < 0.05).

Abbreviation: cCAF, circulating cancer‐associated fibroblast.

### 3.5 CAFs and Molecular Phenotype of Tumor Cells in Tumor Microenvironment

**FIGURE 5:**
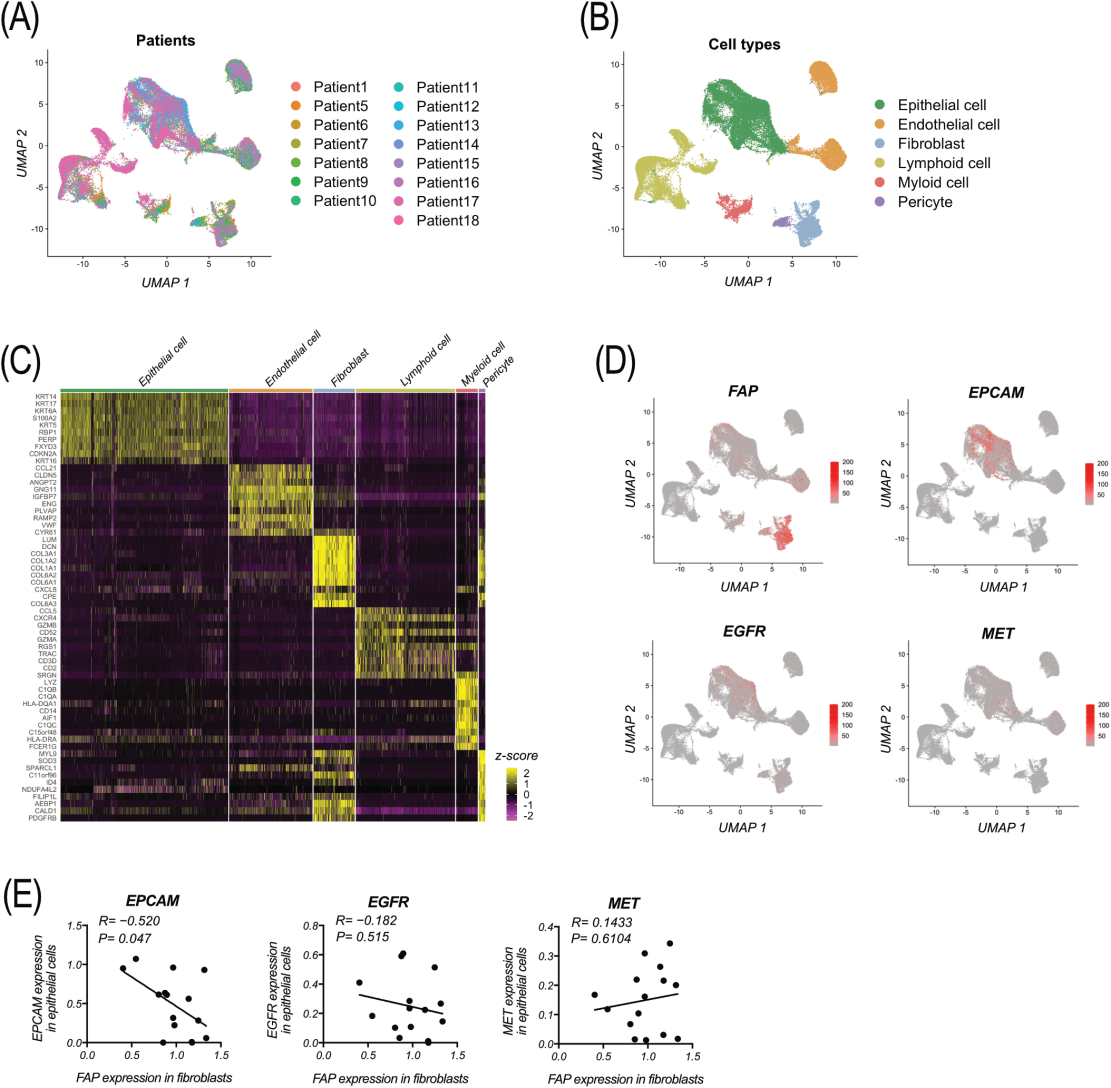
Single‐cell RNA sequencing (scRNA‐seq) of CD45‐negative cells isolated from HNSCC tissues. scRNA‐seq data of CD45‐negative cells isolated from 15 HNSCC tissues were analyzed. (A) UMAP showing the distribution of samples. (B) UMAP showing six different cell types. (C) Heat map showing the top differentially expressed in each cell type. (D) UMAP showing the expression of *FAP*, *EPCAM*, *EGFR*, and *MET*. (E) The correlation between *FAP* expression in fibroblasts and *EPCAM*, *EGFR*, and *MET* expressions in epithelial cells. UMAP, Uniform Manifold Approximation and Projection. [Color figure can be viewed at wileyonlinelibrary.com]

Finally, we analyzed the relationship between CAFs and the expression of three epithelial‐related genes in tumor cells within the tumor microenvironment using a public database. We analyzed scRNA‐seq data of CD45‐negative cells obtained from 15 HNSCC samples. The distribution of samples is visualized using UMAP (Figure 5A). Cells were then clustered, and six different cell types (epithelial cell, endothelial cell, fibroblast, lymphoid cell, myeloid cell, and pericyte) were identified by differentially expressed genes in each subcluster (Figure 5B,C). The top three gene expressions in each cell type were visualized using UMAP (Figure S2). Moreover, *FAP*, *EPCAM*, *EGFR*, and *MET* were confirmed to be expressed on fibroblasts or epithelial cells (Figure 5D). Notably, *FAP* expression in fibroblasts was inversely correlated with *EPCAM*, but not *EGFR* or *MET* expression in epithelial cells (Figure 5E), suggesting that in the tumor microenvironment, *EPCAM* expression in tumor cells may be regulated by CAFs.

## 4 Discussion

The objective of this study was to detect CAFs in peripheral circulation in patients with HNSCC and to explore the complex interplay between cCAFs and CTCs. While we have been able to achieve this objective to some extent, the following three aspects are of particular importance: (1) cCAFs were detected in peripheral blood in patients with HNSCC using molecular detection techniques; (2) the patients in whom cCAFs were identified had a significantly higher proportion of patients with *EPCAM*‐positive CTCs; (3) CTCs coexisting with cCAFs revealed significantly lower levels of *EPCAM* expression compared to those without coexisting cCAFs.

So far, although CAFs in peripheral blood have been paid less attention than those in the tumor microenvironment, their presence and clinical implications have been reported using various methods in several types of cancer, including breast, lung, and prostate cancers [17, 19, 22]. Ao et al. have demonstrated that cCAFs in peripheral blood were detected using a size‐based microfilter technology and double staining of FAP and α‐SMA [17]. Meanwhile, Booijink et al. have also been able to identify cCAF by flow cytometry using EpCAM and FAP [22]. In this study, cCAFs were able to be detected by a combination of negative selection method of CD45‐positive cells and mRNA expression analysis of *FAP*. Although the expression of *ACTA2*, a well‐known marker for CAFs, was correlated with the number of spiked cells and was significantly higher in patients with cCAFs compared to those without cCAFs, it was highly likely that its expression includes gene expression in other contaminated cell populations, and therefore may be more useful as an auxiliary or subset marker rather than as an identifying marker for cCAFs. Future validation using visual methods such as immunofluorescence may further substantiate the presence and morphology of cCAFs. From a clinical perspective, cCAF‐positive patients had a significantly higher incidence of lymph node metastasis. CAFs in the tumor microenvironment contribute to a multistep process in the development of cancer such as metastasis, immunomodulation, stemness maintenance, and treatment resistance [12, 23]. Based on these findings, it has already been reported that CAFs and/or CAF‐derived secretory proteins promote lymph node metastasis in esophageal, oral cavity, and bile duct cancers [24, 25, 26]. Similar to CAFs in the tumor microenvironment, cCAFs in HNSCC may also function to support the establishment of lymph node metastasis through interactions with circulating tumor cells. Meanwhile, our data failed to show any significant association with distant metastasis or prognosis. To date, in breast cancer, patients with metastatic cancer have been reported to have a higher incidence of cCAFs compared to patients with localized cancer [17]. Similarly, circulating fibroblast‐like cells were only detected in 58.3% of patients with metastatic prostate cancer, but in none of the patients with localized prostate cancer [27]. Moreover, elevated cCAF numbers correlated with poorer clinical outcomes in various cancer patients diagnosed at the metastatic stage. The discrepancy in these results may be due to the small sample size, differences in the targeted diseases or disease stages, and different cCAF‐detecting techniques. To further clarify the clinical significance of cCAFs, a larger scale study targeting the same disease is needed.

As expected, the patients in whom cCAFs were identified had a significantly higher proportion of CTC‐positive patients. More interestingly, *EPCAM* expression in CTCs derived from patients with cCAFs was significantly lower than that from patients without cCAFs. As survival mechanisms in the circulation, CTCs formed not only homoclusters among themselves, but also heteroclusters through interaction with other types of cells such as immune cells, endothelial cells, and CAFs [18, 28, 29]. Our findings suggest that clustering with cCAFs triggers EMT in CTCs, resulting in reduced *EPCAM* expression in CTCs. Thus, the presence of cCAFs provides the opportunity for cluster formation with CTCs, and CAFs have been shown to alter the properties of CTCs to be favorable for cancer progression, including EMT, tumor dedifferentiation, and acquisition of cancer stem cell‐like traits, as well as their survival. When this phenomenon was verified using scRNA‐seq data from 15 HNSCC samples, the relationship between CAFs and *EPCAM* expression in tumor cells within tumor tissues was found to be consistent with that in peripheral blood. Similarly, in colon cancer cells, coculture with CAFs has been demonstrated to strongly expand cell types showing lower expression of epithelial genes, including *EPCAM* [30].

The most important limitation of this study is the lack of consideration of the heterogeneity of CAFs. Growing evidence indicates that CAFs are heterogeneous populations of various origins and are classified into various subtypes with different functions and different expression markers depending on cancer types [23, 31, 32]. Based on similarities in CAF subtypes across malignancies, at least four subtypes—myofiboblastic CAFs, inflammatory CAFs, antigen‐presenting CAFs, and matrix CAFs—are identified, and no single marker is expressed in all CAFs [33, 34]. It remains to be further elucidated whether the cCAFs identified in the circulation belong to CAFs shed from tumor tissues or to CAFs from bone marrow‐derived progenitors, what their functional differences are, and which CAFs contribute to clinical outcomes. In addition to cCAFs, other nonmalignant tumor‐associated cells such as circulating endothelial cells and cancer‐associated macrophage‐like cells have also been reported in the bloodstream of cancer patients [35, 36, 37, 38]. While not the focus of this study, these rare populations may also contribute to systemic tumor biology and deserve further investigation.

Taken together, our findings suggest that cCAFs exist in the peripheral blood of patients with HNSCC and may contribute not only to the survival of CTCs, but also to tumor progression through changes in the characteristics of CTCs. Further elucidation of cCAFs would provide new insights into the development of biomarkers and therapeutic targets in HNSCC.

## Author Contributions

Conception and design and manuscript writing: **Kazuaki Chikamatsu**; data acquisition: **Hideyuki Takahashi**, **Shota Ida**, **Hiroe Tada**, and **Miho Uchida**; data analysis and interpretation: **Kazuaki Chikamatsu**, **Hideyuki Takahashi**, **Shota Ida**, **Hiroe Tada**, **Masaomi Motegi**, and **Yuichi Tomidokoro**. All the authors have read and approved the final manuscript.

## Ethics Statement

This study was approved by the Ethics Committee of Gunma University Hospital (HS2017‐152). Written informed consent was obtained from all patients.

## Consent

The authors have nothing to report.

## Conflicts of Interest

The authors declare no conflicts of interest.

## Supporting information


**Figure S1** Kaplan–Meier survival analysis in patients with HNSCC. (A) Progression‐free and (B) overall survival based on the presence or absence of cCAFs. (C) Progression‐free and (D) overall survival for three groups: CAF‐positive and CTC‐positive, CAF‐negative and CTC‐positive, and CAF‐negative and CTC‐negative.

**Figure S2** Additional data to Figure 5. UMAP showing the expression of top three DEGs in each cell type. UMAP, Uniform Manifold Approximation and Projection.

Table S1

## Data Availability

The data that support the findings of this study are available from the corresponding author upon reasonable request.
