# Supplementary figures and images for: Detection of Circulating Cancer‐Associated Fibroblasts in Head and Neck Squamous Cell Carcinoma and Their Impact on Circulating Tumor Cells
Source: Head Neck. 2025 Jul 5;47(11):3141–9. doi: 10.1002/hed.28239 (PMC12541681; doi:10.1002/hed.28239)

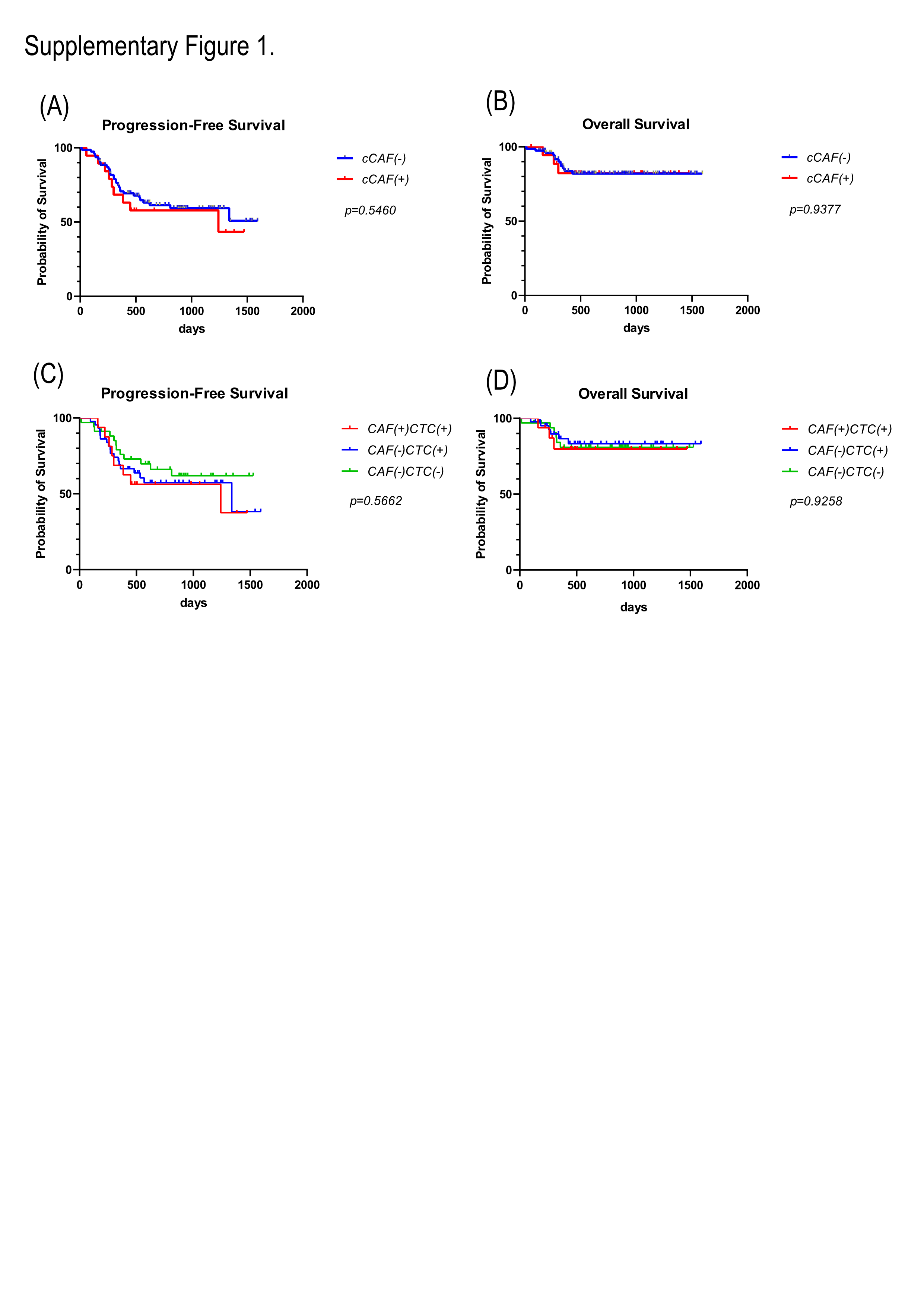

Supplement: Supplementary file 1 — Figure S1 Kaplan–Meier survival analysis in patients with HNSCC. (A) Progression‐free and (B) overall survival based on the presence or absence of cCAFs. (C) Progression‐free and (D) overall survival for three groups: CAF‐positive and CTC‐positive, CAF‐negative and CTC‐positive, and CAF‐negative and CTC‐negative. [file HED-47-3141-s002.tiff]
